# Endoplasmic Reticulum Stress: Its Role in Disease and Novel Prospects for Therapy

**DOI:** 10.6064/2012/857516

**Published:** 2012-12-23

**Authors:** Axel H. Schönthal

**Affiliations:** Department of Molecular Microbiology and Immunology, Keck School of Medicine, University of Southern California, 2011 Zonal Avenue, HMR-405, Los Angeles, CA 90033, USA

## Abstract

The endoplasmic reticulum (ER) is a multifunctional organelle required for lipid biosynthesis, calcium storage, and protein folding and processing. A number of physiological and pathological conditions, as well as a variety of pharmacological agents, are able to disturb proper ER function and thereby cause ER stress, which severely impairs protein folding and therefore poses the risk of proteotoxicity. Specific triggers for ER stress include, for example, particular intracellular alterations (e.g., calcium or redox imbalances), certain microenvironmental conditions (e.g., hypoglycemia, hypoxia, and acidosis), high-fat and high-sugar diet, a variety of natural compounds (e.g., thapsigargin, tunicamycin, and geldanamycin), and several prescription drugs (e.g., bortezomib/Velcade, celecoxib/Celebrex, and nelfinavir/Viracept). The cell reacts to ER stress by initiating a defensive process, called the unfolded protein response (UPR), which is comprised of cellular mechanisms aimed at adaptation and safeguarding cellular survival or, in cases of excessively severe stress, at initiation of apoptosis and elimination of the faulty cell. In recent years, this dichotomic stress response system has been linked to several human diseases, and efforts are underway to develop approaches to exploit ER stress mechanisms for therapy. For example, obesity and type 2 diabetes have been linked to ER stress-induced failure of insulin-producing pancreatic beta cells, and current research efforts are aimed at developing drugs that ameliorate cellular stress and thereby protect beta cell function. Other studies seek to pharmacologically aggravate chronic ER stress in cancer cells in order to enhance apoptosis and achieve tumor cell death. In the following, these principles will be presented and discussed.

## 1. Introduction

The endoplasmic reticulum (ER) is a vital organelle present in all eukaryotic cells. It consists of interconnected, branching membranous tubules, vesicles, and cisternae that provide a distinct subcellular compartment with a number of functions. The rough ER is studded with ribosomes on its outer surface and plays a key role in protein synthesis and secretion. The smooth ER lacks associated ribosomes and therefore is not primarily involved in protein synthesis, but is central to the synthesis of fatty acids and phospholipids, assembly of lipid bilayers, metabolism of carbohydrates, and regulation of calcium homeostasis. In the liver, enzymes in the smooth ER metabolize and detoxify hydrophobic chemicals, such as drugs and carcinogens, and direct them for secretion from the body. While some cells may have little smooth ER, all eukaryotic cells have conspicuous amounts of rough ER, as the latter is essential for the synthesis of plasma membrane proteins and proteins of the extracellular matrix. Rough ER is particularly abundant in secretory cells, such as antibody-producing plasma cells, insulin-secreting beta cells, or cells of milk-producing glands, where a large fraction of the cytosol is occupied by rough ER. The sarcoplasmic reticulum is a specialized form of the ER in muscle cells and functions to sequester and release large amounts of calcium to effect muscle contractions and relaxation [1]. 

The ER is a highly dynamic organelle, and its complex functions can be significantly influenced by a multitude of parameters both inside the cell and in its microenvironment. For instance, the availability of oxygen (hypoxia) or glucose (hypoglycemia), hyperthermia, acidosis, calcium levels, the redox milieu, energy levels (modulated by hypoxia and hypoglycemia), and other factors can impact and disturb proper functioning of the ER, resulting in ER stress and impacting protein folding in the lumen of the ER. Protein folding is a complex process that depends on the interaction of chaperone proteins, foldases, and glycosylating enzymes, as well as appropriate calcium levels and an oxidizing environment. ER stress impairs this process and results in the accumulation of unfolded or misfolded proteins, which leads to the activation of a specific cellular process called the unfolded protein response (UPR) [2, 3]. Activation of the UPR represents the defining criterion of ER stress, although oftentimes the terms UPR and ER stress are used interchangeably. 

The accumulation of unfolded, misfolded, insoluble, or otherwise damaged proteins can irreparably damage cellular functions and thus pose a proteotoxic threat to the survival of the cell. Several cellular mechanisms coordinately function to ameliorate this risk. Prime among these is the core function of the UPR, which is aimed at correct protein folding and overall proper protein processing. However, terminally misfolded proteins, that is, those that cannot be repaired, will be removed from the cell's inventory by one of two separate processes. One is ER-associated degradation (ERAD), which exports damaged proteins back into the cytoplasm and delivers them to the proteasome for degradation and clearance. The other is aggresome formation, where damaged proteins are compacted together with other cellular debris into juxtanuclear complexes and then recycled via autophagy. Indeed, autophagy, a cellular mechanism for the recycling of surplus or defective cellular components, has been found reciprocally linked to ER stress. Several studies have shown that severe ER stress activates the autophagic process and, conversely, that blockage of autophagy leads to aggravated ER stress and cell death [4–8]. (The molecular details of the autophagic process have been covered in recent reviews [9–12] and will not be presented here.) 

A well-documented ultrastructural response to ER stress is displayed by the pronounced dilation of the ER lumen. For example, yeast cells expand their ER volume at least 5-fold under UPR-inducing conditions, and similar effects have also been confirmed in mammalian cells *in vitro* and *in vivo* [4, 13–16]. This expansion of ER lumen is thought to be necessary to accommodate increasing amounts of lumenal constituents, in particular those that are being synthesized to manage ER stress, and thus appears to be an adjustment to cope with increasing crowding conditions, which favor protein aggregation and are detrimental for proper protein folding [17]. 

**Figure 1:**
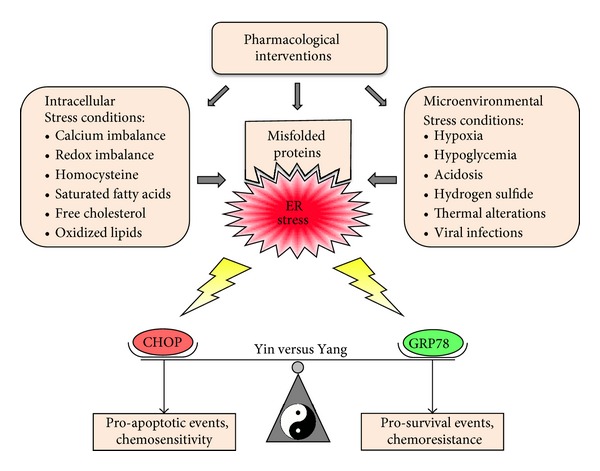
Triggers of ER stress and the *yin-yang* balance of cell survival versus cell death. A great variety of conditions and pharmacological compounds can disturb ER homeostasis, leading to ER stress and the accumulation of unfolded and misfolded proteins. In response, ER stress signaling pathways stimulate pro-survival efforts to either neutralize the stressful insult or adapt to it. GRP78 plays a key role in the cell's attempt to adapt and survive. In contrast, if ER stress is too severe, the pro-apoptotic module of this cellular system gains dominance and shifts the balance towards cell death. CHOP represents a central executor of this latter process. In essence, these opposing processes of cell death versus survival are reflective of the *yin-yang* (shadow and light) concept of Chinese philosophy, where seemingly contrary forces are interconnected and interdependent as part of a greater whole. Although many other components participate in balancing the cell's *yin-yang* response to ER stress, the opposing efforts of prosurvival (*yang*) GRP78 and proapoptotic (*yin*) CHOP represent important tenets of this struggle; as well, their expression levels are being used as convenient markers and readouts as to the ER stress status of cells. Details of GRP78 and CHOP functions are presented in Figures 2 and 3, respectively.

Due to the cytotoxic risk that the accumulation of misfolded/unfolded proteins poses to the cell, it is not surprising that cellular sensors and pathways have evolved to respond to this threat. This stress response system, called the ER stress response or the UPR, displays a dichotomic *yin-yang* characteristic (Figure 1), where mild or short-term stress triggers activation of a response module that either leads to the neutralization of the initial stress or adaption to it, but where severe or long-lasting stress favors activation of a proapoptotic module that will lead to cell death. In both cases, the initial signal (e.g., accumulation of misfolded proteins inside the ER) is transmitted across the ER membrane, through the cytoplasm, and into the nucleus, where alterations in gene expression patterns effect the respective resulting phenotypic outcome, that is, adaptation and survival or apoptosis. 

## 2. Key Players in the ER Stress Response

Considering the diversity of functions of the ER, which include quality control and secretion of proteins, lipid and membrane biosynthesis, and control of intracellular calcium homeostasis, it is not unexpected that a large number of regulatory components participate in these processes and, in one way or another, become involved in the ER stress response/UPR. For example, the ER lumen is rich in calcium-dependent molecular chaperones, such as glucose-regulated protein 78 (GRP78, also called BiP: immunoglobulin heavy chain-binding protein [18, 19]), GRP94, calnexin and calreticulin, enzymes involved in posttranslational protein modifications, such as protein disulphide isomerase (PDI), oxidoreductases, and those performing protein glycosylation and lipidation, as well as numerous others involved in lipid and membrane biosynthesis. 

A variety of disturbances can interfere with proper processing in the ER and thus trigger the ER stress response/UPR (Figure 1). For instance, under conditions of low glucose supply (hypoglycemia), N-linked glycosylation of proteins is impaired [20, 21]. Imbalanced cellular redox homeostasis, which can be caused by hypoxia and prooxidant or reducing agents, interferes with disulphide bonding of proteins [22]. Aberrant calcium levels impinge on the activity of calcium-dependent chaperones [23, 24]. Impaired removal and degradation of terminally misfolded proteins (by blocked ERAD or compromised autophagy) results in the accumulation of these potentially proteotoxic proteins [5, 25–27]. Viral infections may overload the ER lumen with virus-encoded proteins [28–30]. A diet high in fats and sugars (chronic hyperlipidemia and hyperglycemia) has also been linked to increased ER stress, particularly in the liver and in insulin-secreting *β*-cells of the pancreas (see the following) [31–34]. Key players of the ER stress system that are involved in responding to these threats to cellular homeostasis are presented in the following subsections. 

### 2.1. Master Regulator GRP78

Among the many ER-resident proteins, the chaperone GRP78 stands out because in addition to its calcium binding and protein processing function, it exerts a key role as a master initiator of early ER stress/UPR signaling. As implied by its name, GRP78 initially has been characterized as a glucose-regulated protein, where restricting the availability of glucose in cell culture medium resulted in pronounced stimulation of GRP78 transcription and translation, and thus provided initial clues as to its activation during cellular stress conditions [35, 36]. A large number of subsequent studies established that a great variety of cellular and microenvironmental disturbances, as well as many pharmacological interventions, can lead to increased GRP78 expression, along with aggravated ER stress. Indeed, the significantly increased amount of GRP78 protein over baseline expression has become an established indicator and marker for the presence of cellular ER stress [37–39]. 

GRP78 belongs to the heat shock protein 70 (HSP70) family of proteins, where many of its members have been characterized as chaperones within the ER. In recent years, however, it was discovered that GRP78 can also be present outside the ER; for example, the protein was found in the cytosol [40], in mitochondria [41], in the nucleus [42], and at the cell surface of tumor cells [43–47]. It has thus emerged that GRP78, as well as a few other traditional ER-localized chaperones, can function beyond this compartment and are involved in processes not directly connected to posttranslational protein processing [39, 48]. For purposes of this current paper, the focus shall remain on the intra-ER functions of GRP78. 

**Figure 2:**
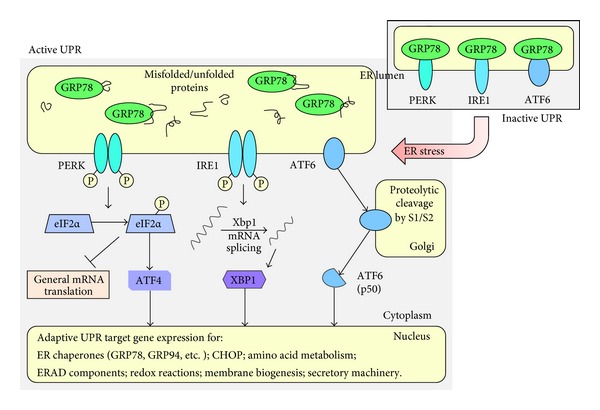
Overview of the three signaling branches of the ER stress response/UPR. In the absence of ER stress, ER luminal GRP78 associates with ER transmembrane proteins PERK, IRE1, and ATF6 to block their activation (shown as inactive UPR in the top right square). Upon ER stress, accumulating unfolded and misfolded proteins inside the ER sequester GRP78, thus dissociating this master regulator from all three transmembrane sensors and relieving their blockage. Activation of PERK entails homodimerization and autophosphorylation, leading to phosphorylation of eIF2*α*, which terminates global protein translation, but exempts selected ER stress-associated proteins, such as ATF4. Activation of IRE1 also entails homodimerization and autophosphorylation. Endonuclease activity of activated IRE1 removes an intron from Xbp1 mRNA to generate a shorter splice variant that encodes transcription factor XBP1. ATF6 translocates to the Golgi, where it is proteolytically cleaved by S1 and S2 proteases to generate the transcriptionally active p50 fragment. All three transcription factors, ATF4, XBP1, and ATF6-p50 translocate into the nucleus where they regulate the expression of a variety of gene products collectively involved in managing ER stress. (See text for further details and references.)

In unstressed cells, a fraction of ER-luminal GRP78 is bound to three different ER transmembrane proteins: (i) inositol-requiring kinase/endoribonuclease 1 (IRE1) [49], (ii) protein kinase activated by double-stranded RNA (PKR)-like ER kinase (PERK) [50], and (iii) activating transcription factor 6 (ATF6) [51]. Binding of GRP78 to the ER-luminal domains of these proteins keeps their activity suppressed and maintains them in an inactive state. Upon ER stress and concomitant accumulation of misfolded and unprocessed proteins, GRP78 is sequestered away from PERK, IRE1, and ATF6 in order to attend to the increased need for protein folding. As a result, dissociation from GRP78 leads to the activation of all three of these transmembrane proteins, thereby unfolding three distinct branches of the ER stress response/UPR (see Figure 2 and general reviews [2, 3, 52]). Among the consequences of these signaling events is increased expression of GRP78, which not only serves to provide the needed additional chaperone capacity, but also eventually will reassociate with PERK, IRE1, and ATF6 in order to return these signaling modules to their inactive modes when homeostasis has been reestablished. 

### 2.2. The IRE1 Signaling Branch

Activation of IRE1 represents the most conserved signaling branch of the ER stress response/UPR [53, 54]. It is a bifunctional enzyme with serine/threonine protein kinase and endoribonuclease (RNase) activity in its cytosolic domain. Release from suppression by GRP78 triggers its homodimerization and autophosphorylation as part of the activation process [52]. Activated IRE1 cleaves a 26-base fragment from the mRNA encoding X box-binding protein 1 (XBP1), resulting in spliced XBP1s and translation of a potent transcription factor controlling the expression of genes involved in ERAD and protein folding, as well as others directing the synthesis of phospholipids that are required for the expansion of ER membranes during ER stress [49, 55]. IRE1 signaling and XBP1 splicing are particularly important in highly secretory cells where the protein folding machinery is continuously engaged with a high amount of nascent proteins [56]. Therefore, this branch of control serves as a key adaptive mechanism to match ER folding capacity with the demands of protein folding [57, 58]. 

**Figure 3:**
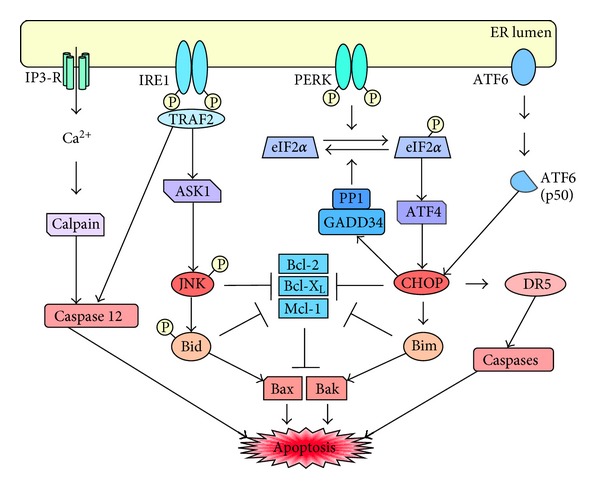
Cell death signaling by the ER stress response/UPR. In case of severe and sustained ER stress, a number of proapoptotic events begin to dominate and lead to apoptosis. Transcription factors ATF4 and ATF6-p50 stimulate CHOP expression. On one hand, CHOP stimulates expression of GADD34, which associates with PP1, resulting in dephosphorylation of eIF2*α*, thus reactivating global cellular protein synthesis. On the other hand, CHOP inhibits antiapoptotic proteins of the Bcl-2 family and stimulates pro-apoptotic Bim, altogether leading to heterodimerization and activation of pro-apoptotic Bax and Bak. CHOP also stimulates expression of cell surface death receptor DR5, which sensitizes cells to pro-apoptotic stimuli, presumably via calibrating the extrinsic apoptotic pathway involving caspase 12. Similarly, activated JNK complements the pro-apoptotic efforts of CHOP. JNK becomes phosphorylated and activated by protein kinase ASK1 upon association of TRAF2 with activated IRE1. Association of TRAF2 with activated IRE1 also leads to activation of caspase 12. Calcium release from the ER via IP3 receptors can activate calpains, which further stimulate caspase 12 activation via proteolytic cleavage of its inactive procaspase precursor.

In addition to splicing a number of mRNAs, a second function of IRE1 is to activate a signaling cascade involved in controlling cell fate with regard to cell death. Here, activated IRE1 recruits tumor-necrosis- factor-receptor- (TNFR-) associated factor 2 (TRAF2), which results in the downstream activation of apoptosis signal-regulated kinase 1 (ASK1) and c-Jun N-terminal kinase (JNK) [59, 60]. On one hand, sustained JNK activity during prolonged ER stress inhibits antiapoptotic members of the Bcl-2 (B cell lymphoma 2) family of proteins. On the other hand, JNK phosphorylates and activates proapoptotic BH3-only proteins, such as Bid (BH3 interacting domain death agonist) and Bim (Bcl-2-interacting mediator of cell death). Combined, these events lead to oligomerization of Bax and Bak, resulting in permeabilization of the outer mitochondrial membrane and execution of the intrinsic apoptotic process [58, 61, 62] (see Figure 3). 

### 2.3. The ATF6 Signaling Branch

ER transmembrane-localized ATF6 harbors a basic leucine zipper (bZIP) motif and transcription factor properties. Upon its release from ER-luminal GRP78, Golgi localization sequences are unmasked, whereupon ATF6 translocates to the Golgi apparatus. Here, it is proteolytically cleaved by Golgi-resident site-1 protease (S1P, a serine-protease) in its ER luminal domain and by site-2 protease (S2P, a metalloprotease) within its region that spans the Golgi phospholipid bilayer, resulting in the release of the cytosolic bZIP transcription factor domain from the Golgi membrane [63]. Upon translocation to the nucleus, ATF6 stimulates expression of a number of genes whose protein products contribute to protein folding, protein secretion, and ERAD, thereby supporting the cell's effort to cope with ER stress and accumulated misfolded/unfolded proteins [37, 64] (Figure 2). Examples of ATF6-regulated genes include GRP78 and GRP94, protein disulphide isomerase (PDI), XBP1, and CHOP [52, 65, 66].

Besides ATF6, a number of other ER-transmembrane bZIP transcription factors have been described in recent years that are also regulated by intramembrane proteolysis. In contrast to ubiquitous ATF6, expression of these factors appears to be tissue specific to variable degrees. Examples are cAMP responsive element-binding protein 3 (CREB3, also called Luman), CREB4/TISP40, CREBH, CREB3L1/OASIS, and CREB3L2/BBF2H2 (see detailed references in reviews: [31, 67]). Although most of these have been found activated upon ER stress and appear to contribute to the general ER stress response/UPR, it is not entirely clear what sets them apart from ATF6 and why cells would require multiple ATF6-like molecules in the ER. It has been surmised that these variant proteins perhaps respond to tissue-specific conditions of ER stress that may require tissue-specific gene expression patterns to resolve that stress [31].

### 2.4. The PERK Signaling Branch

Activation of PERK involves its homodimerization and autophosphorylation, which is followed by phosphorylation of its main substrate, eukaryotic initiation factor 2 alpha (eIF2*α*). Phosphorylation of eIF2*α*  attenuates global protein synthesis, thereby decreasing protein influx to the ER in support of resolving the cytotoxic threat from accumulated misfolded proteins [68]. At the same time, phosphorylation of eIF2*α*  changes the efficiency of AUG initiation codon usage and leads to the preferential translation of a small number of mRNAs, including activating transcription factor 4 (ATF4), a transcription factor that stimulates a set of genes involved in supporting recovery and adaptation [50]. Among ATF4-regulated genes is the one encoding CHOP, a key transcription factor that is important to initiate the apoptotic program in case of excessive ER stress [69] (see details in next subsection). 

Besides eIF2*α*, nuclear factor-erythroid 2-related factor 2 (Nrf2) represents a second immediate substrate for phosphorylation by PERK. Upon activation, this basic-leucine zipper transcription factor migrates to the nucleus where it activates genes encoding antioxidant proteins and detoxifying enzymes [70]. Because ER stress may involve the accumulation of reactive oxygen species (ROS), thereby promoting a state of oxidative stress, Nrf2 plays a critical role in fighting such perturbations in redox homeostasis [71]. The importance of this defensive role of Nrf2 has been further emphasized by findings that Nrf2-deficient cells displayed greatly increased cell death following exposure to ER stress [72]. 

### 2.5. Proapoptotic CHOP

The expression levels of CHOP (C/EBP homologous protein, also called GADD153: growth arrest and DNA damage inducible gene 153 [73, 74]) are kept very low in nonstressed cells. Upon acute ER stress, however, CHOP expression is strongly stimulated through IRE1- and PERK-mediated signaling and the activities of ATF4 and ATF6 transcription factors. The full proapoptotic effect of CHOP only emerges when ER stress cannot be subdued by the efforts of the prosurvival module of the response system, and the levels of misfolded proteins remain high. In this case, CHOP stimulates a transcriptional profile that facilitates a pro-apoptotic program. It includes expression of proapoptotic Bim and repression of antiapoptotic Bcl-2 [75, 76], which represents a mechanism that is aligned with similar pro-apoptotic efforts of JNK mentioned previously (see Figure 3, and detailed refs. in [62, 69, 77]). As well, CHOP induces death receptor 5 (DR5), which further sensitizes cells to apoptotic stimulation by a variety of conditions that cause ER stress [78]. 

 Another target gene of CHOP is growth arrest and DNA damage inducible protein 34 (GADD34), a regulatory subunit of protein phosphatase type 1 (PP1); CHOP-induced stimulation of GADD34 expression leads to PP1 activation and dephosphorylation of eIF2*α*, resulting in the resumption of general translation and normal functioning [79, 80] (Figure 3). Thus, while sustained elevation of CHOP expression triggers strong pro-apoptotic signaling, its initial effect on GADD34 may contribute to the restoration of homeostasis—with the caveat that the renewed supply of client proteins to the ER, if taking place too early, that is, under conditions where ER stress is not yet completely resolved, can trigger the generation of reactive oxygen species (ROS) with deleterious consequences for cell survival [77]. In any case, the dissolution of ER stress entails mandatory suppression of CHOP levels as a prerequisite for return to homeostasis [81]. 

## 3. The *Yin-Yang * Principle of ER Stress

The primary goal of the ER stress response/UPR is to reestablish cellular homeostasis either by eliminating the stressful trigger (e.g., via restoring redox or calcium balance) or by adapting to it (e.g., via permanently increasing its folding capacity in case of revved-up protein synthesis). However, if these countermeasures prove unsuccessful and severe imbalances persist, the response system abandons its prosurvival efforts and instead initiates proapoptotic mechanisms that gain dominance and eventually will lead to cell death. Because of these dichotomic efforts between cell survival and cell death, the ER stress response mechanisms can be viewed as a cellular display of *yin-yang* principles, where the two opposing forces of cell death and survival balance each other for the greater good of ensuring survival of the organism as a whole (Figure 1). 

GRP78, the previously introduced master regulator of the ER stress response, represents the perhaps most critical proponent of the prosurvival *yang* module of this system. As mentioned, GRP78 protein is key in activating the response system in an initial effort to pursue adaptation and cellular survival. Even more so, the robust pro-survival potency of this protein provides significant growth advantage to tumor cells and endows them with the ability to withstand and even thrive under otherwise adverse microenvironmental conditions, such as hypoglycemia and hypoxia that is common within tumor regions with insufficient blood supply. Worse for a patient with cancer, chronically elevated expression of GRP78 in tumor tissue may provide resistance to chemotherapy and may spell worse prognosis [82–86]. 

At the other end of this *yin-yang* balance are proteins such as CHOP and IRE1-regulated JNK, which are representatives of the pro-apoptotic *yin* mechanisms of the stress response. Both proteins exert significant pro-apoptotic efforts in a variety of ways, central among them the suppression of important antiapoptotic proteins and the stimulation of pro-apoptotic components [69, 87] (Figure 3). 

The relevance of IRE1-mediated JNK activation for ER stress-induced cell death has been highlighted by experiments where the activity of this module was blocked with small molecule inhibitors or the use of knockout cell lines [59, 69, 87, 88]. For example, besides modulating the balance of Bcl-2 protein family members, IRE1-JNK signaling can also induce autophagy [6]. As well, IRE1 engages pathways involving stress kinase p38, extracellular signal regulated kinase (ERK), and nuclear factor kappa B (NF-*κ*B) (see details in reviews [31, 58, 89, 90]). 

Activated by the PERK/ATF4 axis and by ATF6, transcription factor CHOP in particular represents a crucial executor of the *yin* module via its ability to impinge on mitochondrial events that function to integrate and amplify the cell death pathways [69, 91, 92]. In unstressed cells, CHOP protein levels generally are below detection levels, but are substantially increased upon acute ER stress. In fact, the presence of conspicuous amounts of CHOP protein represents a marker for the acute phase of the activated ER stress response. Prolonged, high-level expression of CHOP indicates that the ER stress response system has exceeded the limits of its protective *yang* capacity and that it has switched to its pro-apoptotic *yin* module—despite the continued presence of elevated GRP78 [69, 81]. Conversely, during moderately intense short-term stress, or when the cell is adapting to longer-lasting chronic stress, efforts of the pro-survival *yang* module include the suppression of CHOP expression as a prerequisite for recovery and survival, which entails reassociation of GRP78 with and inactivation of the ER transmembrane signaling components PERK, IRE1, and ATF6 [81, 93, 94].

Taken together, pro-survival (*yang*) GRP78 and pro-apoptotic (*yin*) CHOP and JNK represent key opposing forces of the ER stress response. Although many additional components mediate the respective final outcome of this process, in simplified terms the antagonistic activities of GRP78 and CHOP signify the cell's *yin*-*yang* struggle during its efforts to cope with ER stress. For this reason, both proteins are convenient and indeed frequently used markers to distinguish between the chronic and acute phases of ER stress [81, 91, 95].

## 4. Targeting ER Stress for Therapy

As presented in greater detail later, the activated ER stress response system has been found involved in a number of human diseases and therefore is being recognized as an emerging target for therapy (see Table 1). In accordance with the previously described *yin-yang* principle of this cellular system, two conceptually opposing approaches are offered in order to therapeutically target ER stress. On one hand, experimental efforts are aimed at supporting the pro-survival *yang* modules and/or blocking the pro-apoptotic *yin* components in order to subdue the pro-apoptotic processes and achieve increased cellular survival. This approach is appropriate in cases where severe ER stress threatens proper organ function, as is the case, for example, in type 2 diabetes where chronic ER stress may lead to the destruction of pancreatic *β*-cells (see below).  Table 2 shows a list of compounds with inherent potency to ameliorate ER stress and minimize its apoptotic consequences. 

On the other hand, efforts to further aggravate preexisting ER stress and enhance pro-apoptotic processes could be beneficial in the case of malignant neoplasms. For example, pharmacological enhancement of chronic ER stress in cancer cells may exceed the protective *yang* capacity and may trigger selective tumor cell death [96]. The basis of this principle lies in the observation that many tumor cells, unlike normal cells, harbor chronically activated pro-survival ER stress components, such as elevated levels of GRP78, in order to manage intensified protein synthesis or to adapt to hostile microenvironmental conditions, such as hypoglycemia, hypoxia, acidosis, or chemotherapy [38, 86, 95, 97]. As a consequence of the already engaged ER stress system, fewer contingencies may be left to accommodate additional intensities of ER stress in these cells [25, 96]. In comparison, normal cells without chronic ER stress may harbor greater reserves to withstand intervention with pharmacological agents aimed at aggravating the ER stress response (see below).  Table 3 shows a list of compounds that are known to aggravate ER stress and have shown pro-apoptotic potency in tumor cells *in vitro* and in animal models* in vivo*. 

In the following sections, considerations for targeting the *yin-yang* principle of ER stress for therapeutic purposes will be presented. On one side, diabetes will be discussed as an example of a disease where the alleviation of ER stress might hold promise for therapy. On the other side, cancer will serve as an example where the opposite approach, namely, the further aggravation of preexisting ER stress, is being explored for therapeutic purposes. 

## 5. ER Stress in Obesity and Diabetes

The pathogenesis of a number of human diseases has been associated with ER protein-folding defects and ER stress/UPR (Table 1). Perhaps the most convincing evidence for the relevance of ER stress to disease development and progression has been collected in the case of type 2 diabetes (T2D), where the combination of *in vitro* results, animal experiments, and human studies have established ER stress-induced *β*-cell failure in the pancreas as among the fundamental etiologies of this disease (see detailed refs. in reviews [31, 98–101]). 

As of 2010, T2D is estimated to affect about 285 million people worldwide and represents a major cause of morbidity and mortality [102]. The disease is characterized by a complex group of metabolic conditions, including inadequate insulin secretion by pancreatic islet cells and/or peripheral insulin resistance, and dysregulated hepatic glucose production. It has been well recognized that hyperglycemia, saturated free fatty acids, and obesity in general are key risk factors for the development of T2D, and these same conditions are recognized triggers of ER stress, particularly in organs such as liver and pancreas [32, 34, 98, 103]. Progression of T2D places increased demands on pancreatic *β*-cells for insulin production in order to compensate for spreading insulin resistance. Augmented processing of proinsulin to insulin in the ER, combined with the increased presence of free fatty acids and glucose, is thought to trigger chronic ER stress. If these conditions are maintained for extended periods, as would be the case in obese patients and people with high-fat/high-sugar diets, chronic ER stress conditions eventually may lead to *β*-cell death, thus initiating a vicious cycle of exacerbated hyperglycemia [104–107]. As a consequence, the progressive decline in pancreatic *β*-cell function and insulin secretion causes impaired glucose tolerance and established T2D, along with reduced *β*-cell mass [108–111]. 

A large number of *in vitro* and *in vivo* studies, as well as investigations in patients with T2D and obese individuals, some of them postmortem [112, 113], have contributed to and confirmed a key contributing role of ER stress in the development of T2D. For instance, it has been shown that free fatty acids, in particular palmitate, activate the ER stress response in *β*-cells, as indicated by the phosphorylation of PERK and eIF2*α*, concomitant inhibition of protein synthesis, activation of IRE1 and ATF6, and overexpression of ATF4 and CHOP [114–119]. As well, high glucose levels have been shown to elevate several ER stress markers in cultured rat islets and *β*-cells [120, 121]. Phosphorylation of insulin receptor substrate-1 (IRS-1) by JNK leads to the inhibition of insulin signal transduction and contributes to peripheral insulin resistance [122]. Similarly, deficiencies in ER stress pathways, such as impairment of the PERK-eIF2*α*  branch or overly active IRE1 signaling, impair folding capacity and insulin processing within the ER and suffice to trigger *β*-cell dysfunction and death [104, 123–125]. Other reports provided evidence that CHOP expression critically contributes to ER stress-induced *β*-cell death under conditions of increased insulin demand [119, 126–128]. 

Studies with genetically obese (*ob/ob*) or diet-induced obese mice revealed elevated levels of PERK and eIF2*α*  phosphorylation, IRE1-mediated JNK activation, and higher amounts of GRP78 in the liver and adipose tissue of these animals, as compared to lean controls [34]. With the use of knockout cells and synthetic inhibitors, it was demonstrated that IRE1, JNK, and XBP1 significantly regulated insulin receptor signaling, thus establishing a critical role of these ER stress response components in insulin action and obesity [34]. In a subsequent paper [129], the same group of investigators provided evidence that this link between ER stress and T2D can be exploited for therapeutic purposes with orally active chemical chaperones. Two compounds in particular, 4-phenyl butyric acid (PBA) and taurine-conjugated ursodeoxycholic acid (TUDCA), which are agents with known capacity to reduce ER stress, alleviated ER stress and resulted in normalization of hyperglycemia and restoration of systemic insulin sensitivity in these obese animals, thus altogether acting as potent antidiabetic agents [100, 129]. 

Similar protective effects were also observed when berberine, an alkaloid that has been part of traditional Chinese medicine (TCM), was administered to diabetic rats [130]. TCM contains a number of herbs and antidiabetic formulas that are usually served as adjuvants to improve diabetic syndromes in combination with routine antidiabetic drugs [131]. Examples are ginseng, garlic, cinnamon, bitter lemon, rehmannia root, dwarf lilyturf tuber, and others. Several of these herbs have shown some potency to improve insulin sensitivity, stimulate insulin secretion, and protect pancreatic islets and have become popular complementary and alternative approaches to the treatment of T2D syndromes [132–134]. While their molecular mechanism of action remains to be established, the example of berberine could indicate that ER stress pathways might be targeted by at least some of these TCMs. 

In other studies, PBA and TUDCA [135, 136], as well as fluvoxamine [137], were shown to act as leptin-sensitizing agents. Leptin is a protein that is primarily synthesized by adipose tissue, and its circulating levels are proportional to the total amount of fat in the human body. Binding of this hormone to receptors in the hypothalamus signals inhibition of food intake and thus provides an important rheostat to prevent overeating and obesity [138, 139]. In recent years, leptin resistance has been documented in the majority of the obese population and has helped to explain the difficulties of obese individuals to control food intake and body weight. Intriguingly, a number of recent papers have linked leptin resistance to ER stress, thus providing yet another aspect to the potential treatment of obesity with ER stress-modifying agents [136, 137, 140, 141].

The close dynamic relationship between ER stress and obesity-linked illnesses, such as insulin resistance and T2D, has also been established in human subjects. For example, several reports have demonstrated increased levels of multiple ER stress markers in adipose tissue of obese subjects, and these markers were significantly correlated with percent body fat and body mass index (BMI) [142, 143]. A study of obese individuals before and after weight loss through gastric bypass surgery demonstrated that the presence of ER stress markers in liver and adipose tissue was significantly diminished after weight loss and metabolic improvement [144]. Previously mentioned ER stress minimizers TUDCA and PBA were also tested in obese human subjects. In one study, TUDCA was shown to increase hepatic and muscle insulin sensitivity as compared to placebo control, although markers of ER stress in muscle or adipose tissue did not change (and liver tissue was not analyzed) [145]. Another study provided evidence that PBA may provide health benefits by ameliorating insulin resistance and *β*-cell dysfunction in overweight or obese subjects [146]. 

In summary, accumulating evidence indicates a role of chronic ER stress in the development of obesity and T2D and other metabolic diseases (Table 1). Keeping in mind that these types of diseases and their interacting mechanisms are complex, ER stress is probably only one of several contributing factors, although apparently a very important one. This raises the prospect that pharmacological interventions aimed at alleviating ER stress may provide therapeutic benefit, and therefore these possibilities are being intensively investigated. Additional studies need to explore the exact molecular mechanisms of compounds such as TUDCA or PBA, their long-term benefit and safety, and their potential interactions with commonly prescribed diabetes medications, such as the biguanide metformin and peroxisome-proliferator-activated-receptor- (PPAR-) activating thiazolidinediones. 

## 6. ER Stress in Cancer 

Cancer cells frequently deal with a set of stressful conditions that are quite different from the metabolic challenges encountered by liver cells or pancreatic *β*-cells in obese individuals. Cancer cells commonly encounter hostile microenvironmental conditions, such as hypoxia, hypoglycemia, and acidosis, which are fairly typical for many tumor types and which are known triggers of ER stress. However, unlike normal liver cells or pancreatic *β*-cells, cancer cells oftentimes have a high proliferative index, which effectively supports the selection of cell variants with genetic or phenotypic changes that enable adaptation to and survival under stressful conditions frequently documented in tumor cell lines and primary clinical samples [147–150]. Prominent among these changes is chronic activation of the protective *yang* module of the ER stress response system, as indicated by the presence of permanently elevated levels of pro-survival GRP78 in most tumor cells [95, 151].

In breast cancer, for example, overexpressed GRP78 is frequently detected in malignant, but not benign, breast cancer tissue and is correlated with poor prognosis for breast cancer patients [83–85]. Unfortunately for the patient, GRP78 not only protects tumor cells from the detrimental impact of a hostile microenvironment, but at the same time also provides chemoresistance. For example, *in vitro* studies have established that increased GRP78 levels protect cancer cells from the cytotoxic effects of several chemotherapeutic agents commonly used in the clinic, such as paclitaxel (Taxol), doxorubicin (Adriamycin), or temozolomide (Temodar) [85, 86, 151, 152]. In fact, overexpression of GRP78 in breast tumor tissue of patients has been shown to be predictive of their resistance to doxorubicin treatment [82–86]. GRP78 also confers chemoresistance to tumor-associated endothelial cells [152, 153] and supports tumor angiogenesis in mouse models of mammary tumor development [82]. Thus, the critical role of GRP78 in shielding tumor cells from suboptimal microenvironmental conditions and protecting them from chemotherapy has been well recognized [95, 154, 155]. 

Pro-apoptotic CHOP, located opposite to GRP78 on the *yin-yang* balance of ER stress (Figure 1), generally is not conspicuously expressed in tumor tissues or tumor cell lines—despite low level, chronic ER stress conditions—because the pro-survival module maintains dominance and GRP78 acts to keep CHOP transcription low [93, 94]. However, if ER stress is acutely aggravated, CHOP transcription will be strongly stimulated, and the duration and extent of this increase has been shown to represent a decisive factor in determining the cells' fate with regard to survival versus death [75, 81]. 

On one side, the presence of chronic ER stress and permanently elevated levels of GRP78 provides a significant survival advantage to tumor cells exposed to sub-optimal microenvironmental conditions. On the other side, this phenotype distinguishes cancer cells from most normal cells, which generally receive plenty of nutrients and oxygen and therefore do not display symptoms of chronic ER stress. Therefore, this differential may represent an opportunity for therapeutic intervention specifically aimed at the already engaged ER stress response and/or overexpressed GRP78 in cancer cells [25, 37, 156]. In particular, one could envision that tumor-specific blockage of GRP78 function and/or strong stimulation of CHOP expression might serve to provide meaningful therapeutic benefit by tilting the *yin-yang* balance of ER stress in favor of its pro-apoptotic module. In the following, the current practical state of this principle will be presented. 

### 6.1. Inhibition of GRP78

Several studies have presented different methods to block GRP78 function *in vitro* and *in vivo*. For example, knockdown of GRP78 expression by antisense oligonucleotides or siRNA approaches were instrumental in establishing GRP78's pro-survival and chemoprotective roles in a variety of cell types [86, 94, 151, 152, 157, 158]. A very different approach was presented with the use of bacterial subtilase cytotoxin (SubAB), a virulence factor of several major bacterial pathogens, such as* Vibrio cholerae, Shigella dysenteriae, Bordetella pertussis,* and certain pathogenic strains of *Escherichia coli* [159]. The catalytic A subunit (SubA) of this toxin was shown to harbor protease function able to cleave GRP78 in a highly specific fashion, where no other cellular target protein could be identified [160]. In an attempt to verify its potential cancer therapeutic value, SubA was fused to epidermal growth factor (EGF) as a targeting vehicle, and this engineered fusion protein was shown to specifically kill tumor cells overexpressing EGF receptor (EGFR) *in vitro* and *in vivo* [161]. Additional studies suggested that, although GRP78 cleavage is necessary to trigger ER stress-induced cell death by SubAB, additional signaling pathways, including Akt, mTOR (mammalian target of rapamycin), MAPK (mitogen-activated protein kinase), and NF-*κ*B might participate in these processes [162–166] (see also the following). 

There is an increasing number of mostly microbial metabolites that have revealed the unique characteristic of being highly cytotoxic to tumor cells only under hypoglycemic culture conditions, that is, when glucose concentrations were lowered to less than 10% of normal or when glycolysis was blocked via the addition of the hexokinase inhibitor 2-deoxyglucose (2-DG). Examples are arctigenin [167], deoxyverrucosidin [168], efrapeptin J [169], analogs of JBIR [170], piericidin A [171], prunustatin A [172], pyrvinium [173], rottlerin [174], valinomycin [175], and versipelostatin [176]. Collectively, these compounds are considered GRP78 downregulators, because they were shown to block the adaptive induction of GRP78 transcription in response to hypoglycemia. It has been surmised that inhibition of GRP78 stimulation by these compounds prevents hypoglycemic cells from mounting their adaptive survival response, thereby leading to selective apoptosis of sugar-craving tumor cells [176]. Several biguanides (metformin, phenformin, and buformin) also were shown to belong to this group of GRP78 downregulators [177], which is intriguing in the context of metformin's use as an antidiabetic medication [178] and its recently recognized potential for cancer risk reduction [179, 180]. 

The isoflavone and soy ingredient genistein, as well as the polyphenolic green tea component (–)-epigallocatechin-3-gallate (EGCG), have been shown to inhibit GRP78 expression or activity, although not all studies were consistent in this regard. For example, on one hand genistein was demonstrated to block increased GRP78 transcription during ER stress, implying muted pro-survival responses [181–183], whereas other studies showed increased GRP78 expression in response to genistein, implying activation of the adaptive pro-survival response [184, 185]. However, in these latter reports pro-apoptotic CHOP expression also was greatly increased, and the overall outcome presented significantly diminished tumor cell survival despite increased amounts of GRP78, signifying dominance of the pro-apoptotic *yin* over the pro-survival *yang* ER stress module. In comparison to genistein, EGCG has not revealed transcriptional blockage of GRP78, but instead was shown to bind to and inhibit the ATPase activity of GRP78 [186], and this effect is being considered as one of the mechanisms mediating green tea's noted ability to sensitize tumor cells to chemotherapeutic treatment [86, 186, 187]. 

Altogether however, the previously presented prospects of selectively blocking GRP78 in future clinical applications, with the intent to achieve chemoprevention, chemosensitization, or other cancer therapeutic outcomes, are complicated by two important aspects. The first of these is presented by the recognition of oftentimes multifaceted properties of some of these GRP78-inhibitory compounds. For instance, both EGCG and genistein have revealed a multitude of other biological effects (for a few examples, see refs. [188–191] for EGCG, and refs. [192–194] for genistein), which makes it quite difficult to unequivocally ascribe any anticancer outcome to the inhibition of GRP78. Metformin also is known to affect a number of different cellular targets [179, 195], including direct inhibition of complex I of the mitochondrial respiratory chain [196, 197]. The complexity of cellular responses to some of these purported GRP78 inhibitors was further underscored by transcriptome analysis, where it was demonstrated that metformin, versipelostatin, or pyrvinium affected the expression of well over 100 different glucose-regulated genes, besides GRP78, when glucose was removed from the medium of cultured cells [177]. Therefore, it has been difficult to ascertain the specificity of many compounds reported as GRP78 inhibitors. 

The second important aspect complicating the selective targeting of GRP78 arises from the emerging recognition that GRP78 is not solely an ER-luminal protein, but also can be found outside the ER and indeed appears to have functions unrelated to the ER stress response. The presence of GRP78 has been reported for the nucleus [42], mitochondria [41], cytosol [40], and the cell surface of many tumor cells [43, 46, 47, 198]. Cell surface localization in particular, where GRP78 has been found associated with important cell growth-stimulatory partner proteins, has been shown to add to GRP78's pro-survival and proliferation-stimulatory repertoire beyond its ER stress functions [44, 45, 199–202]. Therefore, in view of GRP78's varied subcellular localizations and apparently multiple functions, it has been difficult to determine whether outcomes from the inhibition of this protein can be ascribed to effects on the ER stress response or other processes where GRP78 impacts cell growth and survival. However, the application of antibodies that specifically target cell-surface GRP78 has been tremendously useful in beginning to distinguish between these possibilities and in establishing some of the major functions of cell surface GRP78 [43, 203–205]. As well, the previously mentioned SubA subunit of bacterial subtilase cytotoxin has been found to specifically cleave cell surface-localized GRP78 and therefore also represents a valuable tool in this regard [206]. 

It has been shown that ER stress can actively promote cell surface localization of GRP78 [46], and it is possible that the presence of chronic ER stress in tumor cells may represent a major factor for preponderant cell surface GRP78 in tumor cells. It is therefore not entirely clear whether the anticancer effects resulting from GRP78 inhibition can be, or even need to be, clearly separated into those mediated via interactions at the cell surface and those mediated via interference with the ER stress response system. For cancer therapeutic purposes, the overriding consideration would be to achieve chemosensitization and eliminate tumor growth via effective blockage of GRP78 function, no matter its localization. 

### 6.2. Stimulation of CHOP

Based on the *yin-yang* principle of the ER stress response (Figure 1), in order to effect tumor cell death, the complementary tackle to blocking the *yang* function of GRP78 would be to further aggravate ER stress in order to accomplish a dominance of *yin* processes, in particular the overexpression of the pro-apoptotic master executor CHOP. In this context it is noteworthy to clarify the recently coined [207] term ER stress aggravator (ERSA), which denotes any compound capable of exacerbating preexisting ER stress (as present in most cancer cells), as opposed to “triggering” ER stress, which refers to the induction of ER stress from nonstressed conditions (which is generally the case for normal cells). Thus, the process of ER stress aggravation forms the basic principle of exploiting ER stress specifically for purposes of cancer therapy. 

A large number of pharmacological compounds are known to be able to trigger or aggravate ER stress (Table 3). The classical ER stress triggers, such as thapsigargin, tunicamycin, brefeldin A, ionomycin, or mercaptoethanol have been instrumental over the past two decades in investigating and establishing the cellular processes that govern the ER stress response and UPR [208]. More recently, several clinically used drugs have been found to harbor ERSA activity, even though their original indication was entirely unrelated to ER stress. This latter group includes bortezomib (Velcade), celecoxib (Celebrex), and nelfinavir (Viracept) (see the following).

Among the classical ER stress triggers/aggravators, the sesquiterpene lactone thapsigargin is by far the most widely studied. This compound inhibits sarcoplasmic/endoplasmic reticulum calcium ATPase (SERCA), which results in massive leakage of calcium out of its ER storage compartment and severe ER stress [209, 210]. However, potential clinical use of thapsigargin is unfeasible, primarily due to its well-recognized systemic toxicity and its classification as a tumor promoter [211, 212]. Nonetheless, alternative approaches have been developed to circumvent these drawbacks. One such approach entailed the design of a chemically modified molecule, where thapsigargin was coupled to a peptide carrier that is a substrate for prostate-specific antigen (PSA) protease. In mouse models, this prodrug is specifically activated at sites of metastatic prostate cancer and exerts selective anticancer activity [213]. Another thapsigargin-based pro-drug is activated by carboxypeptidase prostate-specific membrane antigen (PSMA) and has demonstrated promising anticancer activity in several mouse xenograft tumor models *in vivo*, with minimal toxicity to the animals [214]. This latter molecule, called G202, currently is being tested in a phase I dose-escalation clinical trial in patients with advanced cancer in the United States. 

The ERSA activities of bortezomib, celecoxib, and nelfinavir were discovered only after these drugs had been approved for clinical use. Bortezomib had been developed as a proteasome inhibitor, and based on this biological activity it was introduced to the market as a treatment of multiple myeloma (MM) and mantle cell lymphoma [215, 216]. Subsequently, several studies established that proteasome inhibition by bortezomib resulted in potent aggravation of ER stress, as indicated by greatly increased expression of ER stress markers, such as GRP78 and CHOP, *in vitro* and *in vivo* [97, 217–219]. Secretory cells, such as MM, appear to be particularly sensitive to killing via proteasome inhibition, presumably because revved-up protein synthesis places extraordinary demands on protein processing and highly active ERAD to remove terminally misfolded proteins [219–221]. As well, the key ER stress signaling component XBP-1 has been found overexpressed in MM, and its dysregulation has been implicated in MM pathogenesis [222, 223]. ER stress induced by bortezomib has been linked to blockage of NF-*κ*B function, increased cellular sensitivity to TRAIL (tumor necrosis factor-related apoptosis-inducing ligand), and caspase-mediated apoptosis via extrinsic death receptor-initiated and intrinsic mitochondrially controlled pathways [224–228]. Thus, altogether there is good evidence that aggravated ER stress represents a central mechanism of bortezomib-induced tumor cell death and that drug-treated cells die due to proteotoxicity. 

Nelfinavir had been developed as an inhibitor of HIV (human immunodeficiency virus) protease [229]. Due to its protease activity, it is thought to also block cellular proteasome activity [230, 231] and thus to elicit pro-apoptotic activities similar to bortezomib, as indicated by the accumulation of polyubiquitinated proteins, increased expression of ER stress markers GRP78 and CHOP, and caspase activation [232–235]. The discovery of nelfinavir's ERSA activity *in vitro* and in mouse tumor models *in vivo* [232, 233] and its potential for chemosensitization [236–238] and radiosensitization [239–241], all of which were characterized a decade after the drug's approval for the treatment of HIV infections, have spurred efforts to repurpose this drug for cancer therapeutic purposes [242, 243]. In the United States, a number of clinical trials are ongoing to establish and verify nelfinavir's usefulness for inclusion in cancer therapeutic regimens. 

Celecoxib belongs to the class of nonsteroidal anti-inflammatory drugs (NSAIDs) and had been developed as a selective inhibitor of cyclooxygenase 2 (COX-2). It has been approved for the treatment of inflammatory conditions and pain and as an adjunct for the therapy of familial adenomatous polyposis (FAP) [244]. Subsequently, however, several additional biological activities and targets of this drug emerged [245–249]. Prime among these was its potency to inhibit the ER calcium pump SERCA, thus aggravating ER stress in a manner similar to thapsigargin [250]. Indeed, a number of reports have demonstrated that calcium release from the ER is the most immediate effect of celecoxib treatment and can be detected within seconds of adding the drug to cells in culture [251–254]. As well, celecoxib-treated cells display transiently blocked general protein translation via PERK-mediated phosphorylation of eIF2*α*  [255], along with greatly increased expression of ER stress markers GRP78 and CHOP *in vitro* and in tumor tissues of drug-treated animals *in vivo* [97, 252–254]. 

Quite intriguingly, it could be demonstrated that celecoxib's main feature, that is, the ability to block COX-2 activity, can be separated from this drug's cytotoxic potency, and a number of structurally related analogs of celecoxib were created that displayed only one or the other function [256, 257]. For example, 2,5-dimethyl-celecoxib (DMC) was shown to have lost the ability to block COX-2, yet it fully preserved cytotoxic potency [97, 252, 257–262]. Similar outcomes were reported with several other celecoxib analogs, such as TT101 [263, 264], CEA [265, 266], and OSU-03012 [256, 266–269]. Conversely, unmethylated celecoxib (UMC) represents an analog with further increased COX-2-inhibitory potency, but much decreased cytotoxic efficacy [270, 271]. Altogether, these reports clearly established that celecoxib's COX-2-inhibitory function was neither able to nor required to induce cytotoxic outcomes, but that COX-2-independent features of the molecule were responsible. Indeed, a series of experiments with celecoxib and celecoxib analogs demonstrated that these compounds' apoptosis-inducing and anticancer effects were most closely aligned with their ability to trigger ER stress via inhibition of SERCA activity [97, 252, 253, 265, 270–274]. A causal relationship to ER stress was confirmed with knockdown experiments, where siRNA to GRP78 increased and siRNA to CHOP decreased cell death in response to treatment of cells with celecoxib or its analogs [97, 252–254, 275]. 

In summary, bortezomib, nelfinavir, and celecoxib are firmly established medications, and their anticancer activity appears to involve, at least in part, aggravation of ER stress. In the case of bortezomib, the optimized pharmacological function is inhibition of the proteasome, which directly relates to ER stress aggravation. Therefore, proteasome-inhibitory activity and ERSA activity emerge in a similar concentration range of the drug, and bortezomib's classification as an ERSA originates from its proteasome-inhibitory activity. However, this is not the case with celecoxib. Here, the optimized pharmacological function is inhibition of COX-2, and ERSA activity merely represents a secondary, COX-2-independent function that requires substantially higher drug concentrations than inhibition of COX-2. It might be for this reason that celecoxib, although efficacious for chemoprevention of colon cancer [276–278], has not revealed impressive anticancer effects when included in therapeutic regimens aimed at advanced cancer types in humans [279–287]. Nonetheless, extensive work with celecoxib analogs, in particular DMC, has revealed that ERSA activity within the celecoxib molecule can be further optimized, as indeed DMC exerts significantly stronger ERSA activity, along with greater cytotoxic efficacy, than its parental compound [97, 252, 270, 271]. As such, DMC and similar compounds represent promising cancer therapeutic drug candidates aimed at the exploitation of ER stress in tumor cells. While phase I clinical trials with OSU-03012 have been initiated, other celecoxib analogs have not yet been considered for clinical investigations.

## 7. Perspective

The ER stress response system/UPR features *yin-yang* principles, characterized by the struggle for dominance between pro-survival and pro-apoptotic modules and their most prominent components, GRP78 and CHOP, respectively. There is now compelling evidence that chronic ER stress and concomitant activation of one or more branches of the UPR are important in the pathogenesis of a number of diseases. As an illustrative example, the link between high-fat/high-sugar diet, ER stress, and dysregulation of pancreatic *β*-cell function has been presented previously. In such cases, therapeutics aimed at ameliorating ER stress by promoting proper protein processing and generally supporting proper ER maintenance may prove useful for prevention and/or therapy. On the other hand, chronic ER stress in cancer may be exploited therapeutically via the opposite approach, namely, via the selective pharmacological aggravation of the ER stress condition in tumor cells. 

A detailed understanding of the consequences of pharmacological interference with ER stress responses in a patient is necessary in order to translate the respective approaches into therapeutic opportunities. Here, our knowledge is still far from complete, and more research is urgently needed. Among the principal challenges is the identification of which ER stress signaling module(s) represent(s) the most promising target in each of the diverse diseases that have been linked to ER stress. As well, future studies should investigate and evaluate the requirements for acute versus long-term medical interventions, potential side effects of such approaches, their interference with other cellular processes and signaling pathways, and possible crosstalk with inflammation and metabolism in general. 

## Figures and Tables

**Table 1:** Human diseases linked to ER stress.

Disease	Linkage to ER stress	References
Type 2 diabetes	(i) Obesity induces ER stress (ii) Hyperlipidemia and hyperglycemia induce ER stress (iii) Free fatty acids (palmitate) induce beta cell apoptosis (iv) Deletion of CHOP improves beta cell function and survival	[31, 34, 98, 119, 127, 142, 288]

Atherosclerosis	(i) Oxidized lipids induce ER stress (ii) Hyperhomocysteinemia induces ER stress (iii) Cholesterol loading induces ER stress-mediated cell death (iv) Reduced plaque necrosis in mice lacking CHOP	[289–295]

Nonalcoholic fatty liver disease	(i) Forced GRP78 expression reduces hepatic steatosis in mice (ii) ER stress activates cholesterol and triglyceride biosynthesis (iii) Hyperlipidemia and hyperglycemia induce ER stress	[296–299]

Alcoholic liver disease	(i) Alcohol induces ER stress	[32, 300, 301]

Heart disease	(i) ER stress contributes to cardiac myocyte apoptosis (ii) Activation of ER stress in infarcted mouse heart (iii) GRP78 and GRP94 protect against ischemic injury	[302–308]

HBV and HCV infection	(i) HBV induces GRP78 and GRP94 (ii) HCV suppresses IRE1/XBP1 pathway	[309–312]

Alzheimer's disease	(i) Mutant presenilin 1 induces ER stress (ii) Mutant presenilin 1 sensitizes to ER stress-induced apoptosis (iii) AD brains show ER stress features	[313–318]

Parkinson's disease	(i) Parkin expression impacts ER stress (ii) ATF4 leads to increase in parkin expression	[317–321]

Huntington disease	(i) Polyglutamine induces ER stress (ii) ER stress markers are present in postmortem HD brain	[322–327]

Amyotrophic lateral sclerosis	(i) Mutant SOD1 activates ER stress (ii) Mutant SOD1 interferes with ERAD (iii) ER stress markers detected in spinal cord of ALS patients	[318, 328–330]

Prion disease	(i) ER stress markers detected in brains affected with prions (ii) ER chaperones are involved in regulation of misfolded prion protein	[331–334]

Cancer	(i) Tumor-specific microenvironment activates ER stress (ii) Cancer cells display chronic display of ER stress markers (iii) Knockdown of GRP78 or of CHOP affects chemosensitivity	[83, 97, 151, 222, 335–337]

**Table 2:** Compounds with potency to ameliorate ER stress.

Compound	Target/effect	References
Chemical chaperones (TUDCA, 4-PBA)	(i) Increased protein folding capacity (ii) Increased ERAD efficiency (iii) Minimized caspase activation	[129, 338–343]

Inducers of chaperone activity (lithium, valproate, BIX)	(i) Increased expression and activity of chaperones (ii) Supportive of cell-protective ER stress mechanism	[344–350]

Benzodiazepines	(i) Inhibition of ASK1 function and IRE1-ASK1 signaling (ii) Obstructive to pro-apoptotic ER stress mechanism	[88]

Inhibitors of eIF2-alpha phosphatase (salubrinal, Guanabenz)	(i) Inhibition of PP1/GADD34 phosphatase activity (ii) Supportive of attenuated global protein synthesis	[351–353]

Antioxidants (BHA, TM2002, and baicalein)	(i) Sequestration of free radicals (ii) Reduction of oxidative stress and apoptosis	[354–357]

Inducers of antioxidant pathways (carnosic acid, triterpenoids)	(i) Stimulation of NRF2 pathway (ii) Protection from oxidative stress and resulting apoptosis	[358–363]

Stress kinase inhibitors (JNK or p38 inhibitors)	(i) Inhibition of pro-apoptotic JNK or p38 pathways (ii) Antagonism to pro-apoptotic CHOP function	[354, 364–367]

BHA: butylated hydroxyanisole.

BIX: BiP/GRP78 inducer X (1-(3,4-dihydroxyphenyl)-2-thiocyanate-ethanone).

4-PBA: 4-phenyl butyric acid.

TUDCA: tauroursodeoxycholic acid.

**Table 3:** Representative compounds with potency to trigger and aggravate ER stress.

Compound	Mechanism linking to induction of ER stress	References
Thapsigargin, celecoxib, DMC	(i) Inhibition of SERCA activity (ii) Ensuing calcium imbalance	[209, 246, 250, 368, 369]

A-23187, ionomycin	(i) Calcium ionophores: stimulation of Ca^2+^ flux (ii) Ensuing calcium imbalance	[208, 370, 371]

Tunicamycin	(i) Glycosylation inhibitor (ii) Ensuing obstruction of protein folding	[372, 373]

2-Deoxyglucose	(i) Hexokinase/glycolysis inhibitor (ii) Ensuing obstruction of protein folding	[208, 374, 375]

2-Mercaptoethanol, Dithiothreitol	(i) Reducing agents (ii) Disruption of disulphide bonds	[376, 377]

Geldanamycin	(i) HSP90 and GRP94 inhibitor (ii) Ensuing impairment of protein folding	[378, 379]

Brefeldin A	(i) ADP-ribosylation factor inhibitor (ii) Impairment of protein trafficking	[380, 381]

Bortezomib, Nelfinavir	(i) Protease and proteasome inhibitors (ii) Ensuing accumulation of terminally misfolded proteins	[217, 219, 230, 233, 382–384]

## Abbreviations

ATF: Activating transcription factor
CHOP: CCAAT/enhancer binding protein homologous protein (also called GADD153)
2-DG: 2-deoxy-D-glucose
DMC: 2,5-dimethyl-celecoxib
DR5: Death receptor 5
ER: Endoplasmic reticulum
ERAD: Endoplasmic reticulum-associated degradation
ERSA: Endoplasmic reticulum stress aggravator
GRP78: Glucose regulated protein of molecular weight 78 (also called BiP)
IRE1: Inositol-requiring enzyme
NF-*κ*B: Nuclear factor kappa B
4-PBA: 4-phenyl butyric acid
PERK: Protein kinase activated by double-stranded RNA (PKR)-like ER kinase (also called pancreatic ER kinase)
SERCA: Sarcoplasmic/endoplasmic reticulum calcium ATPase
TRAIL: Tumor necrosis factor-related apoptosis-inducing ligand
TUDCA: Tauroursodeoxycholic acid.
